# Long-term follow-up of 453 patients with pelvic organ prolapse who underwent transvaginal sacrospinous colpopexy with Veronikis ligature carrier

**DOI:** 10.1038/s41598-020-61995-z

**Published:** 2020-03-19

**Authors:** Chin-Jui Wu, Wen-Chun Chang, Kuan-Ju Huang, Yun-Chiao Hsieh, Lin-Hung Wei, Bor-Ching Sheu

**Affiliations:** 1 Department of Obstetrics and Gynecology, National Taiwan University Hospital, College of Medicine, National Taiwan University, Taipei, Taiwan; 2 Department of Obstetrics and Gynecology, National Taiwan University Hospital Hsin-Chu Branch, Hsin-Chu City, Taiwan

**Keywords:** Risk factors, Ligaments, Outcomes research

## Abstract

Sacrospinous ligament fixation (SSLF) is one of the most utilized surgeries in the management of pelvic organ prolapse (POP). We conducted a large-series study of SSLF in a tertiary center by an experienced urogynecologic team. The 453 women with POP who underwent SSLF at National Taiwan University Hospital in the period from 2002 to 2015 are reviewed. All patients received unilateral SSLF with Veronikis ligature carrier. Concomitant anterior colporrhaphy was performed in 75.3% of the cases and posterior colporrhaphy in 78.6%. The mean operation time was 92.3 ± 31.5 minutes. The intraoperative blood loss was 92.3 ± 91.4 ml. The objective cure rate was 82.5%, and 79 (17.5%) patients recurred. The Kaplan-Meier recurrence-free analysis showed a steep decline during the first postoperative year, and the yearly number of recurrent patients decreased as the follow-up period proceeded. A comparison of the site of recurrence found that anterior compartment prolapse was the most common with 57 cases (12.6%). Paravaginal repair is frequently implemented in the management of recurrent anterior prolapse. In conclusion, SSLF provides excellent support to the apex compartment, and our long-term results show that the anterior compartment is the most commonly encountered type of POP recurrence.

## Introduction

Pelvic organ prolapse (POP) occurs when the female bladder, uterus, vaginal stump, small bowels and/or large bowels descend into the vagina. The prevalence of POP reaches 3–6% as measured by symptoms and up to 50% by vaginal examination^1^. Epidemiologic studies estimate the lifetime risk of undergoing an operation for prolapse or incontinence has increased from 11.1% to 19% from 1997 to 2010^2,3^ Lower urinary tract symptoms (LUTS) such as urinary frequency, infection, or overactive bladder are usually accompanied by POP. Nowadays, surgical treatment remains the mainstay of POP therapy.

Sacrospinous ligament fixation (SSLF) is one of the most utilized surgical procedures in the management of pelvic organ prolapse (POP). It was first developed by Sederl and Richter to fix the vaginal vault at the sacrospinous ligament alone by the vaginal route^4,5^. In 1971, Nichols and Randall introduced sacrospinous ligament fixation in the United States^6^. The objective cure rate is good, ranging from 67% to 100%^7,8^. However, a recent randomized prospective study showed a 5-year failure rate after SSLF of about 70%^9^.

Surgeons have sought new material to improve the long-term outcome. Polypropylene mesh was designed to reinforce weakened native tissue. In the last two decades, the trend of mesh augmentation in POP surgery initially rose steadily but then tapered off and began falling as safety concerns arose and mounted^10^. Finally, in 2019, the U.S. Food and Drug Administration (FDA) ordered all manufacturers to stop selling and distributing surgical mesh intended for transvaginal repair of anterior compartment prolapse because of safety issues. This triggered an alarm against transvaginal mesh use and has put native tissue repair, including SSLF, once again under the spotlight.

Previous prospective studies have indicated that the majority of SSLF recurrence is located in the anterior compartment, also called the cystocele^11^. The 5-year incidence of recurrence reported in long-term studies has ranged from 6 to 29%^12–15^. Until now, few suggestions have been proposed to lessen recurrence after POP surgery. Thus, we conducted this retrospective, large-series study to obtain 2–10-year (mean of 5 years) follow-up outcomes after SSLF in a tertiary center by an experienced urogynecologic team. We aim to describe the long-term results, defining the anatomic failure sites, and develop strategies to manage recurrence.

## Results

### Demographic description

Four hundred seventy-eight patients were enrolled in the study, and 453 patients were included. Two patients were excluded because their SSLF procedures were modified. Three patients were excluded due to their previous suspension surgeries. Another 20 patients were lost in follow-up: 12 because they died, 4 had malignant neoplasm, and the other 4 lost contact. The demographic data of 453 patients are reported in Table 1. The mean follow-up period was 5.5 years, ranging from 24 months to 120 months. The mean age was 64.2 ± 11.13 years old. The median parity was 3, ranging from 1 to 9. The mean BMI was 25.8 ± 3.26. The majority (80.3%) of cases had a pelvic pressure sensation. In addition, 346 patients (76.4%) had urinary symptoms, with increased urinary frequency (42.3%) being the most common, followed by urinary incontinence (28.9%), straining to void (26.7%), feeling of incomplete emptying (15.2%), and urgency (13.6%). In 20.3% of the cases, the previous hysterectomy due to other benign causes was recorded. The majority of patients (52.3%) were categorized in stage III prolapse.

**Table 1:** Demographic data of all patients (n = 453). Abbreviations: BMI, body mass index; POP-Q, pelvic organ prolapse quantification.

	Mean (range)	n (total 453)	Percent (%)
Mean age (years)	64.2 (±11.13)		
Median parity	3 (1–9)		
Mean BMI (kg/cm^2^)	25.8 (±3.26)		
Menopause	50.8 (±4.08)		
Previous hysterectomy		92	20.3%
**Urinary symptoms**			
Frequency		192	42.3%
Incontinence		131	28.9%
Straining to void		121	26.7%
Urgency		62	13.6%
Feeling of incomplete emptying		69	15.2%
Urinary tract infection		9	1.9%
**Prolapse symptoms**			
Pelvic pressure sensation		364	80.4%
Vaginal bleeding		32	7.1%
**Defecatory symptoms**			
Fecal incontinence		7	1.5%
Feeling of incomplete evacuation		6	1.3%
**POP-Q stage**			
I		5	1.1%
II		135	29.8%
III		237	52.3%
IV		76	16.8%

### Surgery

All patients received unilateral SSLF with Veronikis ligature carrier. The concomitant anterior colporrhaphy was performed in 75.3% of the cases and posterior colporrhaphy in 78.6%. An optional vaginal hysterectomy was performed in 53.4% of the patients. Only 6.2% of the patients received paravaginal repair (PVR) at the time of initial operation to correct a lateral defect of the cystocele. The mean operation time was 92.3 ± 31.5 minutes. The intraoperative blood loss was 92.3 ± 91.4 ml. The postoperative pain score by VAS at 6 hours and 36 hours showed a significant decrease, with a *p-*value < 0.0001. The adjunct operation and intraoperative data are summarized in Table 2.

**Table 2:** Operation details (n = 453).

	Mean ± SD	n (total 453)	percent
Sacrospinous ligament fixation		453	100%
Anterior colporrhaphy		341	75.3%
Posterior colporrhaphy		356	78.6%
Vaginal hysterectomy		243	53.4%
Paravaginal repair		28	6.2%
Perioperative blood Loss (ml)	92.3 ± 91.4		
Operation time (min)	92.3 ± 31.5		
Pain Score (VAS) postop 6 hours	3.17 ± 0.9		
Pain Score (VAS) postop 36 hours	1.58 ± 0.5*		

Abbreviations: VAS, visual analog scale.

*P < 0.0001.

### Follow-up and recurrence

**Figure 1:**
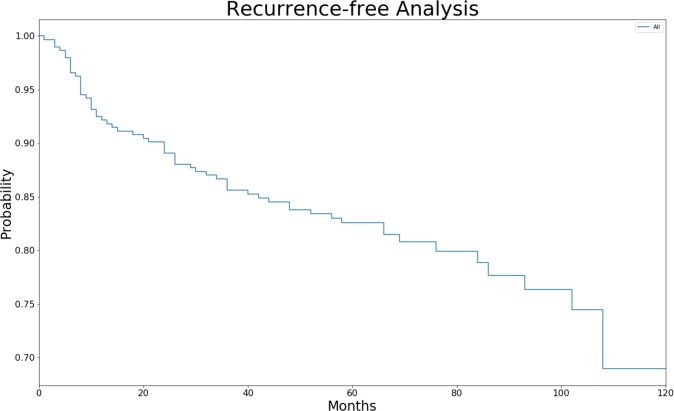
Kaplan-Meier analysis for recurrence-free months. 79 of 453 patients had recurrences in the follow-up period. Recurrence was defined as (1) symptomatic POP-Q stage I, (2) POP-Q stage II or higher prolapse in any compartment. A rapid drop was noted in the first year. A stable downward curve was found from the second year to the 10th year.

The Kaplan-Meier analysis for recurrence of a total of 79 recurrent patients is shown in Fig. 1. Interestingly, there is a steep decline during the first postoperative year. Thirty-four patients recurred in the first year. As the follow-up period proceeded, the annual rate of recurrence became smaller. For all patients, the objective cure rate was 82.5%, with a mean tracking of 65.9 ± 23.9 months. The minimum follow-up was 24 months. The five patients presented solely with urinary incontinence after SSLF surgery were counted separately. They were treated with tension-free vaginal tape (TVT), and no further incontinence was reported.

The recurrent compartments by year are summarized in Table 3. Among the cumulative 10-year follow-up outcomes, 79 (17.5%) of the cases met the criteria of recurrence. Of these 79, 36 (7.9%) were recurrent apical prolapse, 57(12.6%) anterior prolapse, and 26 (5.7%) posterior prolapse. It is worth mentioning that only 36 patients (7.9%) decided to receive a second operation for recurrent prolapse, which is about half of the total recurrent patients. When we followed up these patients, no one experienced or was treated for a second recurrence after the second operation. The re-operation methods for recurrence included repeated SSLF in 19 patients, anterior colporrhaphy in 24 patients, posterior colporrhaphy in 22 patients. There were 21 adjunct PVR operations performed. The adjunct urinary incontinence surgeries included three TVT, one trans-obturator tension-free tape (TOT), and one Burch suspension. These procedures were performed at the same time as the second operation.

**Table 3:** Recurrent compartments by year (n = 79).

Outcome	n	Apex	Anterior	Posterior	Re-operation
1–6 mo	15	7	10	5	9
7–12 mo	19	7	11	7	9
2 y	9	4	8	1	4
3 y	15	4	12	4	5
4 y	7	5	6	2	2
5 y	4	1	2	0	3
6 y	3	2	1	3	1
7 y	2	1	2	1	1
8 y	1	1	1	1	0
9 y	4	4	4	2	2
Total	79	36	57	26	36

Five patients were complicated postoperatively solely by urinary incontinence without prolapse recurrence. Tension-free mid-urethral sling surgery was performed to treat them.

### Risk factors of recurrence

Statistical analysis was performed to analyze the factors contributing to recurrence. Multiple Cox proportional-hazards regression analysis showed that age is the only significant variable (Table 4). The hysterectomy, parity, initial advanced pelvic organ prolapse quantification (POP-Q) stages, adjunct PVR of initial surgery, or other adjunct anterior or posterior colporrhaphy are not significant risk factors in our study.

**Table 4:** Multiple Cox Proportional-Hazards Regression Analysis of Recurrent Risk Factors.

Variables	Hazards Ratio	95% C.I.		p
Age	1.062	1.034	1.092	<0.0001
**Parity**				
<=3	1.00	Referent		
>3	0.827	0.481	1.422	0.4923
**POP-Q Stage**				
II	1.00	Referent		
III	1.393	0.797	2.434	0.2449
IV	1.104	0.531	2.295	0.7901
**Hysterectomy**				
Never	1.00	Referent		
Ever	0.850	0.458	1.578	0.6071
**Anterior colporrhaphy**				
No	1.00	Referent		
Yes	0.584	0.306	1.115	0.1032
**Posterior colporrhaphy**				
No	1.00	Referent		
Yes	0.959	0.504	1.827	0.8996
**Paravaginal repair**				
No	1.00	Referent		
Yes	2.043	0.905	4.610	0.0854

Abbreviations: POP-Q, pelvic organ prolapse quantification.

*p < 0.05.

## Discussion

Our study is one of the most extensive retrospective series of sacrospinous ligament fixation with an average 5-year follow-up. Although Cruikshank *et al*. published a 695-patient series and followed up to 16 years, their patients were divided into three groups according to the evolution of surgical methods. The largest group (Group C) showed that the 301 patients who underwent SSLF and 12 different kinds of adjunct repairs had a mean follow-up of 3.6 year^16^. Our study is of 453 cases that underwent SSLF, among which more than 70% received concomitant anterior and posterior colporrhaphy. Compared with Cruikshank’s study, our study has a higher similarity of surgical treatment, a larger series, and a longer follow-up. Other retrospective studies were either small in sample size or short in follow-up period^8^. A reasonable trend can be seen that the shorter the follow-up period, the higher the cure rates, which indicates the recurrence of POP is time-dependent, and long-term tracking is required.

In our study, we achieved an 82.5% success rate, and the reoperation incidence was 7.9% (36/453). This result is comparable to other SSLF studies tracking more than two years. In our study, we defined recurrence according to symptomatic POP-Q stage I or POP-Q stage II-IV of any clinical compartment, which is compatible with most studies^11,12,15,17^. A previous randomized prospective study (OPTIMAL trial) compared sacrospinous ligament fixation and uterosacral suspension^9^. Their objective recurrent criteria, however, was the strictest one, which defined surgical failure as POP-Q point C descending more than one-third of the total vaginal length or other points beyond the hymen. The strict definition explains why their anatomic cure rate (40.3% in 5 years) was worse than other studies. In the OPTIMAL trial, the Kaplan-Meier survival curves for anatomic failure showed a steep change in the first year, which is similar to our data. We defined the high failure rate in the first year as “technical failure.” Technical failure represents 37.9% of our recurrence. A similar high failure rate can also be found in Cruikshank’s study with 23, 14, and 11 recurrences, respectively, in the first, second, and third year^16^. Other studies either counted all recurrence together or lost cases in the first year^12,14,15^. A possible explanation for technical failure might be improper postoperative care and anchorage failure. Correct postoperative care is necessary for all patients who undergo SSLF and includes not lifting heavy objects. Because suspension of the sacrospinous ligament depends on two sutures on the ligament, improper lifting of excessive weight results in the suture line cutting into the anchorage site, which causes the detachment of the vaginal stump or SSL, and finally leads to recurrence. From our experience, patient education is important to avoid excessive lifting until fibrosis of the anchorage has adequately formed. In contrast to technical failure, the observed long-term failure was more stable. From the second year to the 10^th^ year, a gradually decreasing trend can be seen in Fig. 1. Similar slope curves were depicted in several other studies as well^9,14^. Age and recurrence showed a significant correlation in our study. The reason why the long-term failure occurred is still poorly understood. More studies are needed to investigate the causal mechanism.

The concomitant hysterectomy was as high as 53.4% because hysterectomy was the preferred adjunct surgery to gain access to the operation field in the early period of our study. However, we noticed a trend toward uterus preservation in the later period. A recent meta-analysis showed that the uterus-preserving operation resulted in similar outcomes of prolapse^18^. We showed that concomitant colporrhaphy helped to prevent recurrence under the condition of uterus preservation. The idea of combining these procedures was advocated by other studies^9,16^. Previous prospective studies have indicated the majority of SSLF recurrence is located in the anterior compartment, also called the cystocele^11^. The 5-year incidence of this recurrence reported in long-term studies is 6–29%^12–15^. The existed explanation is that posterior distortion of the vagina leads to pressure on the anterior vagina. In our study, we found a higher rate of PVR in our follow-up surgeries to repair recurrence, which indicates that repair of a lateral defect of the cystocele is crucial. The study by Cruikshank *et al*. also suggested the importance of lateral defect repair, and they incorporated PVR in treating their latest group C patients^16^. A possible mechanism might be that the lateral distortion of the vagina caused by a unilateral SSLF induces pelvic pressure on the other side. A right-side SSLF might aggravate the left side cystocele over time. However, statistical significance between recurrence and either a lateral defect of the cystocele or apical failure was not observed, perhaps because both tend to coincide. According to Dr. Delancey’s opinion, 60% of cystocele size is explained by apical descent, while the other 40% is not^19^. In addition, for the re-operation cases, the patient and doctor both desired a complete repair of all compartments. Therefore, we think reconstructive surgery should view the pelvis as a whole dynamic unit. Repairing one unit might cause pressure to another. The suspension of the apex should be accompanied by the corresponding repair of weak units.

In our series, we chose PVR and anterior colporrhaphy to reinforce the recurrent cystocele. This is possibly because the number is small, with only 28 cases (6.2%) and their severity is also higher. Reviewing the literature, Dr. Benson chose bilateral sacrospinous ligament fixation to avoid recurrence on the opposite^20^. A newer approach employing bilateral SSLF plus paravaginal repair with mesh achieved a high cure rate in a pilot study^21^. The concept of paravaginal repair comes from the lateral defect of cystocele. Dr. Delancey reported a high prevalence of paravaginal defects of up to 87–88% in cadavers and an associated detachment of the arcus tendinous fascia pelvis (ATFP)^22^. For preoperative diagnosis, 3D ultrasound or MRI can aid in the detection of a lateral defect, according to a recent review^23^. We believe that our approach is based on the observation and intuition of experienced surgeons. In our data, no second recurrence was noted after re-operation.

Sacrospinous ligament fixation provides excellent support to the apex compartment. The long-term cure rate is as high as 82.5%, and the recurrent pattern usually involves the anterior compartment. First-year failure and long-term failure are different and should be interpreted separately. Advanced age showed a tendency to recur, with a significant *p*-value < 0.05. Paravaginal repair for a recurrent anterior prolapse may help. A preoperative diagnosis and concomitant site-specific repair may be beneficial to the long-term outcome of sacrospinous ligament fixation.

## Materials and Methods

Our study is a retrospective study. Based on our formal process and procedure, general consents were normally obtained from the patients and we have obtained the approval from the institutional review board (IRB) and the ethics committee which approved our research. From 2002 to 2015, women with POP who underwent SSLF in the National Taiwan University Hospital were reviewed. Patients with a history of previous suspensory surgery such as sacrocolpopexy, transvaginal mesh augmentation, or SSLF were excluded. Patients who had been diagnosed with cancer during the follow-up period were also excluded. All methods in this study were performed in accordance with the relevant guidelines and regulations.

Patients presenting objective prolapse by pelvic examination and symptoms of POP such as pelvic pressure sensation, urinary frequency, straining to void, or defecatory symptoms were suggested to receive surgery. In the patient series, all received SSLF as their main surgery. Concomitant anterior and/or posterior colporrhaphy or vaginal hysterectomy were non-compulsory procedures recommended according to vaginal examination and patient symptoms.

The SSLF surgery was performed with Veronikis ligature carrier and Miya hook, which was described in detail in previous studies^24,25^. Non-compulsory vaginal total hysterectomy was usually performed before SSLF. In certain clinically indicated cases, anterior and posterior colporrhaphy was performed after SSLF. The patients were kept in the hospital for 3 to 5 days postoperatively. Pain scores were regularly recorded during the period of admission, and the voiding function was evaluated. Under a stable recovery condition, patients were discharged to an out-patient department for follow-up. During the first year, the follow-up period was monthly at first and then every three months. Stable patients were evaluated every six months after the first year. The recurrence or failure of the reconstruction surgery was defined as: (1) symptomatic POP-Q stage I or (2) POP-Q stage II or higher prolapse in any compartment, according to the International Continence Society definition^26^. The term “symptomatic POP-Q stage I” means that the patient had concurrent bulging symptoms and objective POP stage I. The bulging symptoms included a subjective downward sensation at the pelvis or bulging feeling from the vagina. Solely urinary symptoms, however, are not defined as sacrospinous colpopexy recurrence or failure.

Re-operation was performed in a site-specific manner. For apical recurrence, a repeated SSLF was usually performed. For anterior recurrence, anterior colporrhaphy with or without paravaginal repair (PVR) was performed. The decision was made according to the surgeon’s physical examination of the defect site. To diagnose a para-vaginal defect, the patient should be in the lithotomy position. We used two long smooth forceps to apply to the vaginal wall with each tip of the forceps is held against the ischial spines to imitate the paravaginal support from the arcus tendineus fascia pelvis (ATFP). The patient was asked to perform maximal Valsalva, and if no prolapse was observed, the prolapse was said to be paravaginal or lateral defect^27^. If a lateral defect was identified, PVR was performed in addition to anterior colporrhaphy. The surgical procedures of PVR were as follows: after dissection of the anterior compartment, a finger was inserted to confirm the paravaginal defect, and to ATFP or arcus tendineus levator ani (ATLA). Two or three vaginal Deaver retractors were used to create exposure. Permanent sutures were used in a 3-point closure incorporating the pubocervical fascia, arcus tendineus, and vaginal wall (For detailed surgical method, please see the ref. ^28^). If no specific defect could be palpated, solely anterior colporrhaphy was completed. Posterior prolapse was repaired by posterior colporrhaphy and perineorrhaphy.

Statistical analysis was performed using SAS software (version 9.4; SAS Institute, Cary, NC). Statistical methods included the Student’s test, Chi-square test, Fisher’s exact test, and multiple Cox proportional-hazards regression analysis. A difference was considered statistically significant when *p*-value is <0.05. The Python “lifelines” package was used to perform the Kaplan-Meier survival analysis.

The data that support the findings of this study are available from National Taiwan University Hospital, but restrictions apply to the availability of these data, which were used under license for the current study, and so are not publicly available. Data are however, available from the authors upon reasonable request and with permission of National Taiwan University Hospital.
